# Effective population size of *Culex quinquefasciatus* under insecticide-based vector management and following Hurricane Harvey in Harris County, Texas

**DOI:** 10.3389/fgene.2023.1297271

**Published:** 2023-11-22

**Authors:** Xinyue Huang, Giridhar N. Athrey, Phillip E. Kaufman, Chris Fredregill, Michel A. Slotman

**Affiliations:** ^1^ Department of Entomology, Texas A&M University, College Station, TX, United States; ^2^ Department of Poultry Science, Texas A&M University, College Station, TX, United States; ^3^ Harris County Public Health, Mosquito & Vector Control Division, Houston, TX, United States

**Keywords:** approximate Bayesian computation, *Culex quinquefasciatus*, population genetics, effective population size, vector control, Hurricane Harvey

## Abstract

**Introduction:** *Culex quinquefasciatus* is a mosquito species of significant public health importance due to its ability to transmit multiple pathogens that can cause mosquito-borne diseases, such as West Nile fever and St. Louis encephalitis. In Harris County, Texas, *Cx. quinquefasciatus* is a common vector species and is subjected to insecticide-based management by the Harris County Public Health Department. However, insecticide resistance in mosquitoes has increased rapidly worldwide and raises concerns about maintaining the effectiveness of vector control approaches. This concern is highly relevant in Texas, with its humid subtropical climate along the Gulf Coast that provides suitable habitat for *Cx. quinquefasciatus* and other mosquito species that are known disease vectors. Therefore, there is an urgent and ongoing need to monitor the effectiveness of current vector control programs.

**Methods:** In this study, we evaluated the impact of vector control approaches by estimating the effective population size of *Cx. quinquefasciatus* in Harris County. We applied Approximate Bayesian Computation to microsatellite data to estimate effective population size. We collected *Cx. quinquefasciatus* samples from two mosquito control operation areas; 415 and 802, during routine vector monitoring in 2016 and 2017. No county mosquito control operations were applied at area 415 in 2016 and 2017, whereas extensive adulticide spraying operations were in effect at area 802 during the summer of 2016. We collected data for eighteen microsatellite markers for 713 and 723 mosquitoes at eight timepoints from 2016 to 2017 in areas 415 and 802, respectively. We also investigated the impact of Hurricane Harvey’s landfall in the Houston area in August of 2017 on *Cx. quinquefasciatus* population fluctuation.

**Results:** We found that the bottleneck scenario was the most probable historical scenario describing the impact of the winter season at area 415 and area 802, with the highest posterior probability of 0.9167 and 0.4966, respectively. We also detected an expansion event following Hurricane Harvey at area 802, showing a 3.03-fold increase in 2017.

**Discussion:** Although we did not detect significant effects of vector control interventions, we found considerable influences of the winter season and a major hurricane on the effective population size of *Cx. quinquefasciatus*. The fluctuations in effective population size in both areas showed a significant seasonal pattern. Additionally, the significant population expansion following Hurricane Harvey in 2017 supports the necessity for post-hurricane vector-control interventions.

## 1 Introduction

*Culex quinquefasciatus*, also known as the southern house mosquito, is widely dispersed throughout tropical and temperate regions on earth. This species originates from West Africa, and its global expansion is primarily due to human activities (Alaniz et al., 2018). *Culex quinquefasciatus* serves as a principal vector of various arboviruses and filarial parasites, such as West Nile virus (WNV), Japanese encephalitis virus, St. Louis encephalitis virus (SLEV), and *Wuchereria bancrofti*. This species plays an important role in the transmission of multiple mosquito-borne diseases as a critical bridge vector, as it can exploit various birds and mammals as hosts and links reservoir or amplifier hosts to humans in urban, suburban, and rural regions (Bhattacharya and Basu, 2016). *Culex quinquefasciatus* is currently one of the most common vector mosquito species in Harris County.

The importance of *Cx. quinquefasciatus* as a disease-causing pathogen vector in the US increased dramatically with the arrival of West Nile virus in New York City in 1999 and its subsequent rapid range extension throughout North America (Ronca et al., 2021). For example, a total of 1,868 cases of WNV were reported during the 2012 WNV outbreak in Texas (Murray et al., 2013). Even during the “regular” year 2014, 13% of the 9,698 pools containing 813,236 *Cx. quinquefasciatus* from Harris County and the Houston area tested positive for WNV (Martinez et al., 2017). Currently, insecticide-based approaches remain effective for mosquito control in the US. However, the rapid selection for insecticide resistance in mosquito populations in the United States has become a threat to the continued success in controlling mosquito-borne diseases, and this is the case in Houston as well (Huang et al., 2023). Against this background, integrated vector management (IVM) has been proposed to use resources optimally, such as reducing unnecessary insecticide applications for vector control. As an important part of IVM, a mosquito surveillance program has been conducted in Harris County since 1965 (Poh et al., 2019). Many studies pointed out that increased mosquito abundance alters the potential risk of mosquito-borne disease transmission, particularly as a consequence of climate extremes (Nosrat et al., 2021) and landscape modifications (Norris, 2004), because these conditions provide for habitats used by vector mosquitoes. Although several factors, such as human density and human-vector contact (Thongsripong et al., 2021), determine vector-borne disease transmission, vector abundance is a crucial component in the transmission of vector-borne diseases.

Researchers have recommended using indicators assessing mosquito abundance to evaluate risk exposure of arboviral disease transmission, including WNV infection (Gu et al., 2008). Current approaches for mosquito abundance estimation are mainly based on adult mosquito sampling data collected by a variety of mosquito traps (Diuk-Wasser et al., 2006), such as Biogents Sentinel trap (BG), the Centers for Disease Control and Prevention (CDC) light trap, and gravid traps (Gorsich et al., 2019). A previous study conducted by Karki et al. (2016) suggested that weather conditions, landscape type, and trapping methods can affect the estimation of mosquito abundance, which is evaluated by the number of mosquitoes collected in light and gravid traps. In population studies, the total population size (also known as census population size, N_c_) is inferred from field surveys. However, N_c_ is not always an informative index of abundance, e.g., in endangered species studies, as it fails to clarify the profound effects of population genetic structure on population size estimates. Given the limitations of calculating N_c_ accurately, estimating the effective population size (N_e_) of a targeted population is preferred in some contexts. N_e_ was introduced as a measure of the evolutionary change caused by random sampling effects (Wright, 1931) and is defined as the size of an idealized Wright-Fisher population (Fisher, 1999) with the same genetic parameters as the practical population under consideration (Crow and Kimura, 2017). Widely used as a fundamental parameter in population genetics and conservation biology, a variety of methods for estimating N_e_, such as the heterozygote excess method, linkage disequilibrium (LD) method, or based on temporal changes in allele frequency (also known as moment estimator and probabilistic method). Each of these methods make specific assumptions about the populations being sampled (Wang et al., 2016). At the same time, the simplified models have their own advantages and pitfalls (Charlesworth, 2009).

Linkage disequilibrium (LD) is a non-random association of alleles within or between loci, which can be another source of information to estimate N_e_. In a large random-mating population without selection, allele frequencies are independent between different loci when in the state of linkage equilibrium. Thus, random genetic drift in a finite population can result in correlations of alleles, which can be informative about the history of a population’s size. In our study, the LD method was used to estimate N_e_ with NeEstimator in addition to the coalescent approximate Bayesian computation approach (Do et al., 2014).

N_e_ estimation based on approximate Bayesian computation (ABC) has emerged as a promising tool for population genetic studies. ABC approaches use multiple summary statistics from genetic samples, and use different signals from the observed data to help infer population trends. The influence of any single summary statistic on the outcome tends to be diminished by increasing numbers of summary statistics. The accuracy and stability of ABC does not always increase with an increasing number of summary statistics used in the analysis due to the curse of dimensionality (Beaumont, 2010). DIYabc is a software package that incorporates comprehensive historical demographic analysis utilizing DNA polymorphism data, including single-nucleotide polymorphisms (SNP) and microsatellites (Cornuet et al., 2014). ABC methods and ABC-based approaches, including DIYabc, have been proven a valuable tool to study N_e_ of mosquito populations and factors that potentially impact N_e_, such as vector control interventions (Athrey et al., 2012; Hodges et al., 2013).

In this study, we estimated the N_e_ for *Cx. quinquefasciatus*, collected in Harris County, Texas and considered three major impacting factors, including vector-control approaches, seasonal weather conditions and extreme climate events. *Culex quinquefasciatus* was subjected to vector control operations using insecticides, including malathion, permethrin, and its synergist piperonyl butoxide (PBO) during 2016 and 2017. The continued efficacy of mosquito control can be threatened by the emergence of insecticide resistance, which has been widely reported in other parts of the world (Raghavendra et al., 2011), including Harris County (Lee et al., 2020; Huang et al., 2023). Therefore, our primary aim was to investigate if the effective population size of the *Cx. quinquefasciatus* population in Harris County, was different between the intensively treated and untreated areas. As is well recognized, *Cx. quinquefasciatus* populations in subtropical areas usually contract during winter due to low temperatures (Bhattacharya and Basu, 2016). Our second aim was to evaluate whether the N_e_ of mosquito populations in both areas of Harris County experienced a bottleneck during the winter season.

Additionally, Hurricane Harvey, a devastating hurricane, made landfall in the Houston area in 2017. The third aim of this study was to explore the impacts of a typical extreme climate event, Hurricane Harvey, on N_e_ of *Cx. quinquefasciatus* populations. The effects of hurricanes on the N_e_ of mosquito populations are complicated; on the one hand, water stagnation following hurricanes offers a breeding ground for mosquitoes, which may result in an expansion of the affected mosquito population (Breidenbaugh et al., 2008). On the other hand, heavy rainfall may flush larvae from their breeding sites or directly kill adult mosquitoes suppressing the mosquito population (Charlwood and Braganca, 2012). Considering these factors, we defined four possible demographic scenarios in the DIYabc analysis, including bottleneck, expansion, decline, or constant effective population size, which will be further described below.

## 2 Material and methods

### 2.1 Mosquito collection

**FIGURE 1:**
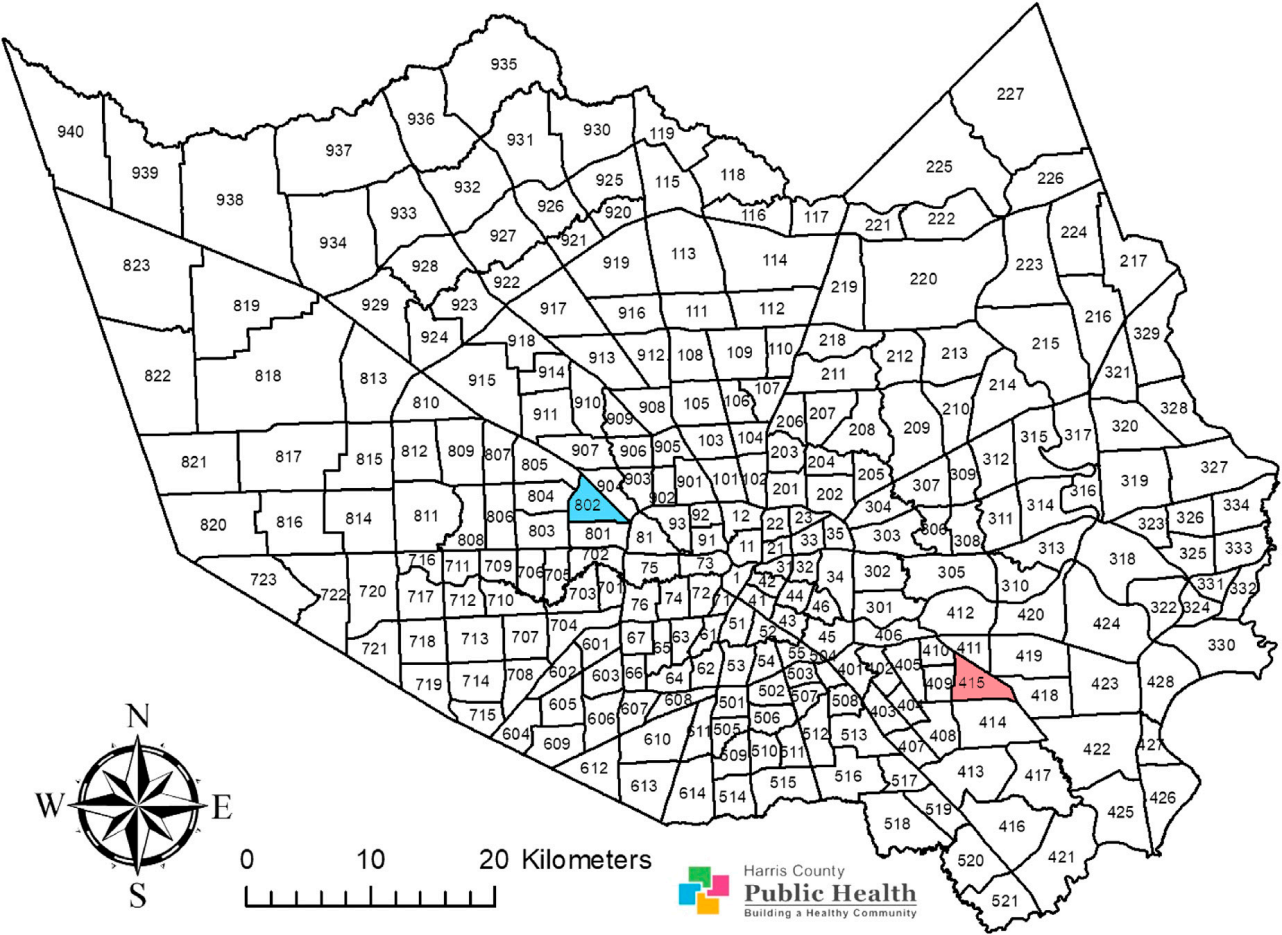
Adult *Culex quinquefasciatus* were sampled from two operational areas, area 415 and area 802, in Harris County, Texas. Area 415 is highlighted in red while area 802 is highlighted in blue. This map shows the Harris County boundary, presented as a map image layer created by PHES_AGO on 7 June 2017, and updated on 7 May 2020. This map also shows the operational area boundaries, presented as a map image layer crafted by PHES_AGO on 4 November 2016, and updated on 9 May 2020. The map layer for county boundary (Map service: Harris County boundary masked) (https://www.arcgis.com/home/item.html?id=a8aa2ef4067348c79ccea62857a2f623) and the layer for Harris County operational area boundaries (MVCD_Operational_Areas) (https://www.arcgis.com/home/item.html?id=66643535e01b42d3aae5d4647f5e1a6c) were generated with ArcGIS (https://www.arcgis.com/home/webmap/viewer.html; ESRI, CA) by Harris County Public Health and are publicly available. There are no special restrictions or limitations on the terms of use of the layers integrated into this map. This map was completed by assembling these two layers and by coloring the research areas using the ArcMap 10.8 software (ESRI, CA).

Adult *Cx. quinquefasciatus* are routinely collected by Harris County Public Health (HCPH) personnel from Houston, Texas, United States. The samples used in this study were collected four times per year in 2016 and 2017 from two operational areas, 415 and 802 (Figure 1). Samples from a single time point were collected within a 1 week time frame (Supplementary Table S1). Mosquitoes were stored at −80 °C until processing. There were no mosquito control operations by HCPH in area 415 during 2016 and 2017. In contrast, 26 malathion spraying operations were performed on ten separate dates, and ten permethrin spraying operations were performed on five separate dates during the summer of 2016 at area 802. Neither site was subjected to adulticidal spraying in 2017. From area 415, mosquito samples collected in weeks 14, 23, 38, and 46 in 2016 and in weeks 2, 20, 32, and 42 in 2017 were included in this study. From area 802, mosquito samples collected in weeks 14, 23, 39, and 48 in 2016 and in weeks 12, 20, 31, and 42 in 2017 were included.

### 2.2 DNA extraction

For each timepoint and location, 95 individuals were included. Total DNA was extracted and purified from each individual mosquito. The whole mosquito body was homogenized using a Qiagen^®^ TissueLyser (Qiagen, Germantown, Maryland). Mosquito tissues were individually transferred into a well of a 96-well plate, which included a negative control. DNA extraction and purification were performed on the BioSprint^®^ 96 workstation (Qiagen, Hilden, Germany) following their standard protocol. The final DNA product was stored at 4°C.

### 2.3 Species diagnostic

Morphological species identification was conducted by personnel from HCPH as the primary filtering for the field-collected adult *Cx. quinquefasciatus*. To confirm the result of morphological identification, species diagnostic polymerase chain reaction (PCR) was performed on each sample (Crabtree et al., 1995), using the *PQ10* and the *CP16* primers. Each 20 µL reaction included 10 µL 2× Thermo Scientific™ PCR Master Mix (Thermo Fisher Scientific, Carlsbad, California), 10 ng *PQ10* primer, 10 ng *CP16* primer, 20 ng template DNA, and nuclease-free water. The PCR program was set up following protocol by Crabtree et al. (1995) and was performed on the Eppendorf™ Mastercycler X50a thermocycler (Eppendorf, Hamburg, Germany). PCR products were visualized on a 2% agarose gel. Those samples that failed to show a clear 698-bp band were not used for microsatellite genotyping and data analysis in the following steps.

### 2.4 Microsatellite genotyping

Eighteen microsatellite loci for *Cx. quinquefasciatus* (Fonseca et al., 2004; Smith et al., 2005; Edillo et al., 2007; Hickner et al., 2010) were amplified in multiplex PCR reactions (Supplementary Table S2). The forward primers were labeled using one of three fluorescent dyes, FAM, HEX and NED. Primers for six loci were multiplexed in a single reaction, with each primer in equimolar concentrations of 0.2 µM. Each 50 µL amplification reaction included 25 µL 2 × QIAGEN^®^ Multiplex PCR Master Mix, 5 µL 10× Primer Mix, 20 ng DNA and nuclease-free water (with 3 mM MgCl_2_ in final concentration). The thermoprofile for the multiplex PCR was: 2 min at 95°C, followed by 30 cycles performed for 30 s at 95 °C; 90 s at 57 °C; and 30 s at 72°C, and a final cycle for 30 min at 72°C. The PCR products were submitted to the DNA Analysis Facility on Science Hill at Yale University for fragment analyses. Genotyping was performed using Genemarker (Hulce et al., 2011).

### 2.5 Data analysis

Microchecker version 2.2.3 was used to check for microsatellite null alleles (Van Oosterhout et al., 2004). Loci CX4, CX10, and CX11 were not included in subsequent analyses, as null alleles were detected in these three loci. Due to low amplification efficiency, data of CX5 from area 802 was also not included in downstream analysis. Microsatellite genotyping data were converted to Genepop format with GenAlEx version 6.503 (Peakall and Smouse, 2012).

**FIGURE 2:**
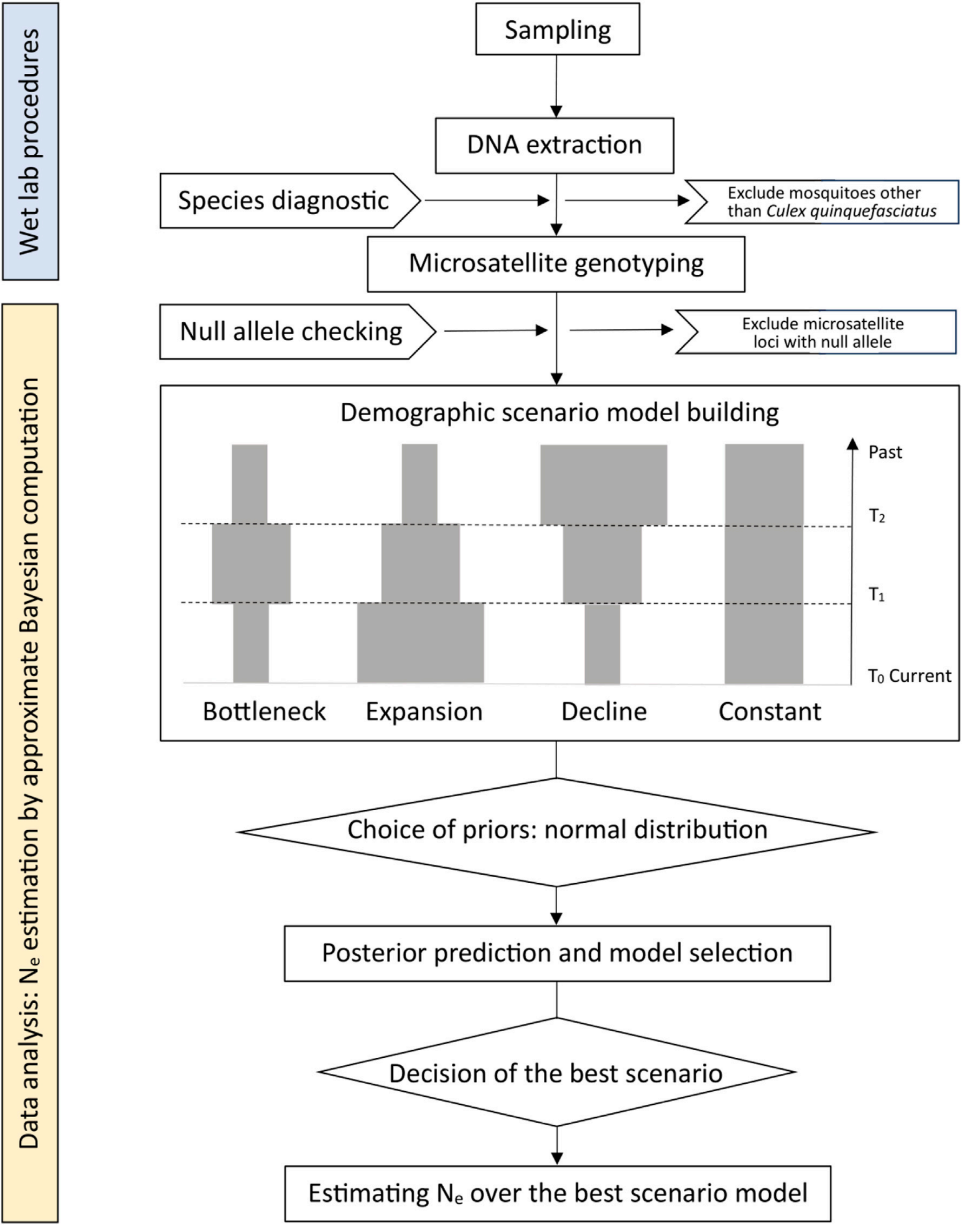
A flow chart of data preparation and analysis. Four historical scenarios include bottleneck, expansion, decline and constant population size. T_0_ is the closest sampling timepoint to the current time and T_2_ is the ancestral sampling timepoint, which is the farthest from the current time.

Data were loaded into DIYabc version 2.1.0 (Cornuet et al., 2014). This software package implements approximate Bayesian computation inferences about population history (Cornuet et al., 2014). We tested four basic demographic scenarios in our study (Figure 2), which included variations of population size (bottleneck, expansion, decline, or constant) for two time periods. For each scenario, 2,000,000 simulated datasets were generated, with specific mutation models and prior distributions for parameters. The summary statistics of each simulated dataset were compared with those of the observed dataset. More specifically, we used three single-sample statistics, including the mean number of alleles, mean genetic diversity (Nei, 1987), mean allele size variance across loci, and three two-sample statistics, including the fixation index (F_ST_) (Weir and Cockerham, 1984), mean index of classification (Rannala and Mountain, 1997; Pascual et al., 2007), and Euclidean distance between every two samples (Goldstein et al., 1995). Through this process, the posterior probability of each scenario was calculated, and the most likely scenario was identified. The effective population size of *Cx. quinquefasciatus* from each site of the Houston area was obtained by estimating the posterior distribution for the most likely scenario.

We set 2 weeks as the average generation time of *Cx. quinquefasciatus*, as the life cycle from egg to adult stage usually takes 10–14 days (Manimegalai and Sukanya, 2014). The minimum value for effective population size (N_e_) was set as 0 and the maximum value for 1,000,000, except for the bottleneck scenario. The prior range for N_e_ at earlier time points was between 0 and 1,000,000, while the prior range of N_e_ at the latter time points was between 0 and 200,000 for the bottleneck scenario.

We tested three time points from spring in 2016 to winter in 2017 per area to find the effects of the winter season. Genotyping data from week 14 and 23 in 2016 and week 2 in 2017 were used for area 415, while genotyping data from week 14 and 23 in 2016 and week 12 in 2017 were used for analysis for area 802. First, we performed the parameter estimation with a prior normal distribution, where parameters had a mean value and a standard deviation within a range between extremum values (minimum and maximum). We also performed the parameter estimation with a uniform prior distribution to test if different prior distributions affect the choice of best demographic scenario. In this case, the prior distribution had minimum and maximum values but without the mean value and standard deviation.

Next, we performed N_e_ estimation from three time points from 2016 and three timepoints from 2017 at area 802 to evaluate the effects of Hurricane Harvey on the N_e_ of the mosquito population. The mosquito samples were collected before and after Hurricane Harvey, which landed in the Houston area on 27 August 2017. For area 802, genotyping data from weeks 14, 23, and 48 in 2016 were used for pre-hurricane analysis in DIYabc, while genotyping data from weeks 12, 20, and 42 in 2017 were used for post-hurricane analysis.

In addition to DIYabc, we used NeEstimator version 2.1 (Do et al., 2014) to estimate the N_e_ of *Cx. quinquefasciatus* from eight sampling time points in 2016 and 2017 at area 802 to further explore the impact of Hurricane Harvey on the N_e_ of mosquito population in Harris County. The N_e_ at each time point was estimated with the Linkage Disequilibrium (LD) method. The mating system was defined as random mating.

## 3 Results

### 3.1 The effects of the winter season and vector control intervention on N_e_ of the Houston mosquito population using DIYabc

The scenario choice did not vary between the uniform or normal distribution; therefore, we present only results based on the normal distribution (Table 1). The estimation for the effective population size and the timing of this change is provided in Supplementary Table S3; Supplementary Table S4. We compared the N_e_ estimation between areas 415 and 802 to explore the impact of vector control intervention, as area 802 was intensively treated by insecticides in 2016 while area 415 did not receive large-scale insecticide treatment by HCPH during 2016 and 2017. However, we did not identify any notable difference. From our examination of the impact of seasonality on *Cx. quinquefasciatus* populations, we found that the winter season had the lowest population sizes as compared to the other seasons examined and typically by more than an order of magnitude (Table 1).

**TABLE 1:** N_e_ parameter estimates generated from the most probable scenarios for the winter season with a prior distribution of Normal distribution.

	Posterior effective population sizes			
Area	Parameter	Median	Mode	95% HDP^a^
415	N_summer16n_	469,000	472,000	309,000—658,000
	N_winter16n_	17,400	1,520	945—149,000
802	N_summer16n_	496,000	520,000	300,000—692,000
	N_spring17n_	104,000	125,000	8,630—194,000

^a^ HDP, highest posterior density.

At area 415, the bottleneck scenario was the most probable, with a posterior probability of 0.9167 (95% CI: 0.8701—0.9632). In this scenario, the median effective population size in the summer of 2016 was estimated at N_summer16n_ = 469,000, whereas the median N_e_ estimate in the winter of 2016 was N_winter16n_ = 17,400 (Table 1). This indicates an approximate 27-fold reduction in N_e_ of during the winter. The estimated timing of population size change, T_11n_, has a median value of 8.5 generations ago, which corresponds to approximately October of 2016. All other scenarios returned very low probability indices.

The most probable scenario at area 802 was also a bottleneck with a posterior probability of 0.4966 (95% CI: 0.4823—0.5109). The second and third most probable scenarios at area 802 were expansion and constant population sizes, with the posterior probability of 0.2578 (95% CI: 0.2451—0.2704) and 0.2457 (95% CI: 0.2335—0.2579), respectively. In the most probable scenario, the median value of N_e_ in the summer of 2016 (N_summer16n_) was 496,000, and the median value of N_e_ in the early spring of 2017 (N_spring17n_) was 104,000 (Table 1). This indicates an approximate 4.8-fold reduction in N_e_ during overwintering in this area. T_12n_ was the estimated time of change in population size, which was estimated to be T_12n_ = 10.2 generations ago approximately in November of 2016 (Table 2).

**TABLE 2:** Time parameter estimates generated from the most probable scenario for the winter season with a prior distribution of Normal distribution.

	Posterior population size changing time (measured by generation)			
Area	Parameter	Median	Mode	95% HDP^a^
415	T_11n_	8.5	8.7	1.2—14.3
802	T_12n_	10.2	10.2	1.9—18.3

^a^ HDP, highest posterior density.

### 3.2 The effects of Hurricane Harvey on Ne of the Houston mosquito population using DIYabc

Hurricane Harvey made landfall in Houston and all of Harris County on 25 August 2017. We compared N_e_ before and after the flooding events at area 802 to study the impact of Hurricane Harvey. We observed a population expansion event following the storm, but it did not differ in magnitude in the most probable population scenario between year 2016 and 2017 using DIYabc.

An increase in N_e_ was observed after June 2016 at area 802. The most probable scenario for the *Cx. quinquefasciatus* population size in 2016 was an expansion with a posterior probability of 0.5320 (95% CI: 0.5005—0.5635). The second most probable scenario in 2016 was that of a bottleneck. It had a posterior probability of 0.2789 (95% CI: 0.2514—0.3064). The median value of N_e_ in the 2016 summer was estimated at N_summer16h_ = 235,000, whereas the median value of N_e_ in the 2016 early winter was estimated at N_winter16h_ = 692,000 (Table 3). This represents a 2.95-fold increase. The sampling time of N_summer16h_ and N_winter16h_ at area 802 were June and November 2016, respectively. T_16h_ was the estimated timing of N_e_ change from N_summer16h_ to N_winter16h_ (Table 4), which was estimated as T_16h_ = 5.8 generations ago, approximately in early September of 2016.

**TABLE 3:** N_e_ parameter estimates at area 802 generated from the best scenario for Hurricane Harvey in the year prior to the hurricane and in the summer/autumn of 2017, which were sample dates just before and just after the hurricane made landfall in the Houston area.

	Posterior effective population sizes			
Year	Parameter	Median	Mode	95% HDP^a^
2016	N_summer16h_	235,000	30,500	15,000—792,000
	N_winter16h_	692,000	917,000	161,000—986,000
2017	N_summer17h_	221,000	37,100	15,100—801,000
	N_autumn17h_	670,000	973,000	139,000—985,000

^a^ HDP, highest posterior density.

**TABLE 4:** Time parameter estimates at area 802 generated from the best scenario for Hurricane Harvey.

	Posterior population size changing time (measured by generation)			
Year	Parameter	Median	Mode	95% HDP^a^
2016	T_16h_	5.8	0.82	0—12.0
2017	T_17h_	6.8	11.0	0—11.0

^a^ HDP, highest posterior density.

An increase in N_e_ was also observed after Hurricane Harvey at area 802 in 2017. The most probable scenario in 2017 was expansion, with a posterior probability of 0.5156 (95% CI: 0.4813—0.5499). The second most probable scenario in 2017 was a bottleneck, with a posterior probability of 0.3402 (95% CI: 0.3065—0.3739). Under the expansion scenario, the median value of N_autumn17h_ before Hurricane Harvey of 2017 was 221,000, whereas the median value of N_autumn17h_ after the hurricane was 670,000 (Table 3). This represents a 3.03-fold increase. The sampling time of N_summer17h_ and N_autumn17h_ at area 802 were May and October in 2017, respectively, and the estimated timing of the change in N_e_ was T_17h_ = 6.8 generation approximately in early August of 2017 (Table 4).

### 3.3 The effects of Hurricane Harvey on N_e_ of the Houston mosquito population using NeEstimator

In contrast to the DIYabc analysis, the N_e_ estimate between data from 2016 to 2017 using NeEstimator were not similar. Overall, the estimated N_e_ value of *Cx. quinquefasciatus* in 2017 was larger compared to N_e_ estimation for the corresponding time period of 2016, suggesting that following Hurricane Harvey a larger mosquito population size was observed than in the previous year.

The four sampling time points of 2016 at area 802 were in April, June, September, and November (Supplementary Table S1). The effective population sizes of these four time points were estimated using NeEstimator at N_Apr16l_ = 246.0, N_Jun16l_ = 316.6, N_Sept16l_ = 712.4 and N_Nov16l_ = 395.0, respectively (Table 5). We observed an increase in N_e_ between June and September during the summer season. The N_e_ then experienced a decline between September and November during the autumn season.

**TABLE 5:** N_e_ parameter estimates generated using NeEstimator to study the effects of Hurricane Harvey on *Culex quinquefasciatus* populations in the Houston area.

	Effective population sizes estimated by LD^a^ method		
Year	Parameter	Estimated population size	95% CI^b^
2016	N_Apr16l_	246.0	181.6—370.5
	N_Jun16l_	316.6	217.4—558.1
	N_Sept16l_	712.4	367.5—6,459.3
	N_Nov16l_	395.0	242.9—950.5
2017	N_Mar17l_	3,837.2	610.8—Infinite
	N_May17l_	1,213.9	450.5—Infinite
	N_July17l_	188.8	149.7—250.6
	Hurricane Harvey made landfall in the Houston area on August 25 in 2017		
	N_Oct17l_	Infinite	Infinite—Infinite

^a^ LD, linkage disequilibrium.

^b^ CI, confidence interval.

For the four sampling time points of 2017 at area 802 in March, May, July, and October (Supplementary Table S1), the effective population sizes of first three time points were estimated at N_Mar17l_ = 3,837.2, N_May17l_ = 1,213.9, and N_July17l_ = 188.8, respectively (Table 5). The value of N_Oct17l_ was estimated as infinite. A decline in N_e_ was observed between March and July 2017. The N_e_ then experienced an increase between July and October, the period during which Hurricane Harvey made landfall in the Houston area on 25 August 2017.

## 4 Discussion

ABC is a simulation-based inference method widely used to examine complex systems (Sunnaker et al., 2013). Since first used by Beaumont et al. (2002), ABC has become a valuable tool for probing the demographic histories of populations, taking advantage of its great flexibility. ABC provides a framework that does not require a specific likelihood function and is therefore suited to the analyses of complex models, also known as scenarios, for which the calculation of model likelihoods is difficult (Lopes and Beaumont, 2010). Instead, the goal of ABC is to calculate the posterior probability distribution of the parameter being investigated (e.g., N_e_) by comparing simulated data with the given empirical data. Previously, Athrey et al. (2012) used ABC to investigate the impact of vector control interventions on malaria mosquitoes, indicating the ability of the ABC approach to study mosquito population demographics.

Here, we used DIYabc to estimate the effective population size (N_e_) of the *Cx. quinquefasciatus* in two areas (802 and 415) of Harris County, TX. Insecticide spraying was applied 16 times at area 802 between June and September 2016, whereas area 415 received none. We hypothesized that these intensive vector control operations influenced *Cx. quinquefasciatus* N_e_. Contrary to our expectation, we failed to detect a difference between areas 415 and 802 in the magnitude of change in N_e_ across the summer. Nonetheless, our analyses generated valuable new insights on the impact of the winter season and a major hurricane on N_e_. We present our discoveries about the effects of the winter season and Hurricane Harvey on N_e_ of *Cx. quinquefaciatus* in Harris County first and then discuss potential limitations of ABC methods.

### 4.1 The impacts of the winter season

Our analyses estimate an approximate 4.8 to 27.0-fold reduction in *Cx. quinquefasciatus* N_e_ during the winter season. A previous study explored the seasonal pattern of mosquito abundance in Harris County using the average number of mosquitoes captured per gravid or storm sewer traps (Poh et al., 2019). This study suggested a large peak of mosquito abundance in May and a second small peak in November, which is consistent with our estimation for overwintering population reduction. Thus, their study, along with ours, indicates that this mosquito overwinters in relatively large numbers in the Houston area, allowing it to build numbers quickly in the spring when conditions become favorable. Whereas the N_e_ prior to the winter was similar in both areas, the N_e_ after the winter season at area 415 (N_winter16n_) was several folds smaller than that of area 802 (N_spring17n_). This could be because the areas could differ in how they can support mosquito breeding during various parts of the year. Furthermore, the sampling time in both areas differed slightly. Finally, perhaps it is possible that extensive vector control at area 415 during summer had a latent effect.

### 4.2 The impacts of Hurricane Harvey

Although our comparison was far from perfect, we were not able to observe an impact of Hurricane Harvey on *Cx. quinquefasciatus.* That is, the increase in N_e_ across the hurricane in 2017 was almost identical to that in 2016, a year without hurricanes in East Texas. The observed increase in N_e_ across the summer months is expected, as is the population size of *Cx. quinquefasciatus* in subtropical areas reaches its peak during the year’s warmest season (Bhattacharya and Basu, 2016). A modeling study by Ahumada et al. (2004) also documented a positive exponential correlation between the population growth rate and temperature.

These ABC results contrast with those obtained using NeEstimator. These analyses suggested a modest 2.2-fold increase from June to September in 2016. In contrast, the population size went to infinite between July and October 2017, indicating a more dramatic population increase in the year Hurricane Harvey hit. Because mosquito larvae are aquatic, heavy rainfall and flooding following hurricanes increase potential developmental sites and therefore results in population expansion (Ahumada et al., 2004; Breidenbaugh et al., 2008).

NeEstimator estimates for 2016 were generally lower than those of 2017, except for N_July17l_. Therefore, we compared the average temperature and precipitation between 2016 and 2017 using the local climatological data from National Oceanic and Atmospheric Administration (NOAA, 2022). The average temperature in 2016 in Harris County was 21.9°C, while the average temperature in 2017 was 22.3°C. The monthly average temperature in 2016 ranged from 11.3°C to 30.6°C while the monthly average temperature in 2017 ranged from 12.1°C to 29.6°C. Overall, the *Cx. quinquefasciatus* population in Harris County experienced a relatively greater temperature fluctuation in 2016 compared to 2017.

Besides temperature, precipitation is another important variable for mosquito propagation. The total precipitation of 2016 in Harris County was 154.8 cm, much lower than the total precipitation of 2017, which was 202.4 cm. The precipitation in 2016 in August was 26.4 cm, but the precipitation in 2017 was concentrated in August, with 99.3 cm of rain recorded in the month. Hurricane Harvey caused concentrated and heavy precipitation in August 2017. The effects of precipitation on mosquito population dynamics have been studied extensively. Recent studies have suggested that temperature and precipitation are necessary for understanding the seasonal patterns of mosquito population dynamics and mosquito-borne disease risks (Beck-Johnson et al., 2017). However, the influence of temperature and natural precipitation is usually complicated, and mosquito population dynamics can be affected by other ecological factors such as irrigation schemes (Sang et al., 2017), overhead tanks, and other structures in urban settings (Thomas et al., 2016), as well as local hydrological conditions (Minakawa et al., 2012). Although the effects of precipitation remain unclear, a previous study by Valdez et al. (2017) suggested that *Cx. quinquefasciatus* abundance decreases with the number of rainy days in a climate with low total rainfall but increases with the number of rainy days in a climate with a high total rainfall. This is reasonable because prior year drought conditions can induce mosquito population increases by suppressing predators (Chase and Knight, 2003). Further analysis is needed to validate the relationship between N_e_ in mosquito populations and potentially influential factors such as temperature and precipitation.

Our results demonstrated that precipitation might have a complicated impact on the N_e_ of mosquito populations, which can become a critical consideration in local vector control strategies. Multiple meteorological variables can affect mosquito abundance and distribution, such as temperature, precipitation, relative humidity, and wind speed (Asgarian et al., 2021). In our study, relatively low rainfall in 2016 and heavy rainfall in 2017 may contribute to mosquito population expansion following landfall of Hurricane Harvey in Harris County. One of the main implications of this work is that routine vector control operations in Harris County can be combined with local dry period to suppress mosquito populations more effectively.

### 4.3 Potential limitations

One major limitation in our study is that we investigated the mosquito N_e_ using samples from two closely located areas in Harris County (Figure 1). There may be gene flow between the mosquitoes collected at area 415 and area 802. As we studied them at relatively small timescales, mosquitoes across these two areas do not belong to an absolutely panmictic population. Therefore, we can distinguish differences between demographics in both areas in this study. However, relatively small time scales can be a concern for N_e_ estimation with ABC approaches. A study on *Anopheles gambiae* populations employed a similar 2-year time scale to estimate N_e_ fluctuation during vector control cycles, which studies mosquito populations within one single region across relatively small time scales (Hodges et al., 2013). They reported a scenario superior to other scenarios with highest posterior probability, which models a N_e_ reduction after each indoor residual spraying round, followed by an increase in N_e_ until the next spray round. Although different vector control approaches and frequencies were applied in this project, their results provided an approach to explore N_e_ fluctuation with similar sampling time interval as our work.

Another major limitation is tied to the selection of demographic models. Using either a very small number of demographic models, or one of the non-representative demographic models, can result in lack of predictive power to decide the best fitted demographic model (Sunnaker et al., 2013). In fact, most model-based studies include either one or a small number of demographic models due to high computational cost for increasing numbers of models in a study. We included four models in our analysis that were the most representative for our study. The ABC method requires massive simulations of large data sets that produce reference tables, and this requires high computational capacity and significant time consumption. The number of simulations required to generate reliable results in the reference table in an ABC analysis is positively correlated with the number of demographic models. This limitation results in researchers needing to describe the models as close to an actual demographic scenario of the studied population as possible, which is difficult without prior knowledge.

Cognizant of these limitations in the coalescent ABC approach implemented with DIYabc, we also used NeEstimator to estimate N_e_ of *Cx. quinquefasciatus*. There are three single-sample N_e_ estimators implemented in NeEstimator, including a linkage disequilibrium method (Waples and Do, 2010), a heterozygote-excess method (Zhdanova and Pudovkin, 2008), and a molecular co-ancestry method (Nomura, 2008). The LD method is a bias-corrected approach with higher precision than the temporal method when N_e_ estimation is performed with microsatellite data (Waples and Do, 2010). However, the LD method provides a more precise estimate for relatively small populations with N_e_ values less than 200 (Waples and Do, 2010). This is much smaller than expected for mosquito populations in Harris County, and it is clear that NeEstimator provided unrealistically low estimates of *C. quinquefasciatus* N_e._

Different types of mosquito traps have different collecting efficacy for male or female mosquitoes. The sex ratio of sampled mosquitoes used in our study may deviate from the actual sex ratio in natural populations. For instance, HCPH collected 720,121 females and 93,115 males in the 2014 investigation using three types of traps, including the CDC gravid trap, CDC miniature light trap, and Biogents sentinel trap (Martinez et al., 2017). In addition, a field evaluation conducted in China revealed that the female-to-male sex ratio of individuals collected by the CDC light trap was 4:1 (Hou et al., 2021). It is worth mentioning that the sex ratio distortion system has been developed and utilized for mosquito control and may affect the sex structure of the field mosquito population (Harris et al., 2012). In this study, we hypothesized a 1:1 sex ratio during N_e_ estimation using DIYabc. As a result, such inaccuracy in sex structure information can introduce bias to the N_e_ estimation results.

In conclusion, our results demonstrated the seasonal fluctuations in the effective population size of *Cx. quinquefasciatus* in Harris County, Texas, including a bottleneck following the winter season. Additionally, our results revealed a population expansion in *Cx. quinquefasciatus* after Hurricane Harvey in 2017. The increase in potential habitats can partly explain the mosquito population growth. We also provide further support for the importance of temperature and precipitation effects on N_e_, providing directions for future research on mosquito population dynamics.

## Data Availability

The microsatellite genotyping data is deposited in the Dryad Repository: http://dx.doi.org/10.5061/dryad.m905qfv76. The original contributions presented in the study are included in the article Supplementary Material, further inquiries can be directed to the corresponding author.
