# Supplementary material for: Effective population size of Culex quinquefasciatus under insecticide-based vector management and following Hurricane Harvey in Harris County, Texas
Source: Front Genet. 2023 Nov 22;14:1297271. doi: 10.3389/fgene.2023.1297271 (PMC10702589; doi:10.3389/fgene.2023.1297271)
Supplement: Supplementary file 1 [file Table1.DOCX]

**Supplementary Table 1. Study sites, sampling years, sampling months and sampling week time points at which estimations of N_e_ were labeled and performed in DIYabc and NeEstimator analysis for adult *Culex quinquefasciatus* collected by the Harris County Public Health agency. Collections without labels in the right three columns were not used in analysis.**

| Sampling Area | Sampling Year | Sampling Month | Week^1^ | N_e_ Label in the DIYabc Analysis for Winter Season | N_e_ Label in the DIYabc Analysis for Hurricane Harvey | Label in NeEstimator |
| --- | --- | --- | --- | --- | --- | --- |
| 415 | 2016 | April | 14 |  |  |  |
|  |  | June | 23 | Ns_ummer16n_/N_summer16u_ |  |  |
|  |  | September | 38 |  |  |  |
|  |  | November | 46 |  |  |  |
|  | 2017 | January | 2 | N_winter16n_/N_winter16u_ |  |  |
|  |  | May | 20 |  |  |  |
|  |  | August | 32 |  |  |  |
|  |  | October | 42 |  |  |  |
| 802 | 2016 | April | 14 |  |  | N_Apr16l_ |
|  |  | June | 23 | N_summer16n_/N_summer16u_ | N_summer16h_ | N_Jun16l_ |
|  |  | September | 39 |  |  | N_Sept16l_ |
|  |  | November | 48 |  | N_winter16h_ | N_Nov16l_ |
|  | 2017 | March | 12 | N_spring17n_/N_spring17u_ |  | N_Mar17l_ |
|  |  | May | 20 |  | N_summer17h_ | N_May17l_ |
|  |  | July | 31 |  |  | N_July17l_ |
|  |  | October | 42 |  | N_autumn17h_ | N_Oct17l_ |

^1^Week of the year
