# Supplementary material for: Effective population size of Culex quinquefasciatus under insecticide-based vector management and following Hurricane Harvey in Harris County, Texas
Source: Front Genet. 2023 Nov 22;14:1297271. doi: 10.3389/fgene.2023.1297271 (PMC10702589; doi:10.3389/fgene.2023.1297271)
Supplement: Supplementary file 2 [file Table2.DOCX]

**Supplementary Table 2. Characteristics of microsatellite markers used in estimation of effective population size for *Culex quinquefasciatus*. Repeat motif and amplicon size (number of base pairs (bp)) refer to the alleles of the sequenced clones. Three fluorescent dyes were used for the microsatellite genotyping, including FAM, HEX and NED. Each primer set includes a forward sequence (F) and a reverse sequence (R) on each side of the microsatellite.**

| Locus ID | Locus Name | Repeat Motif | Amplicon Size (bp) | Dye | Primer sequences (5′−3′) |
| --- | --- | --- | --- | --- | --- |
| CX1 | *C177CA1*^1^ | (CA)_12_ | 130 | FAM | F: CGTTTGCTTCTCGCACCTCA |
|  |  |  |  |  | R: CAGCACAAACATCATCAGGGA |
| CX2 | *C32AC1*^1^ | (AC)_11_ | 184 | FAM | F: CGATGCCTTCCGCAAGATC |
|  |  |  |  |  | R: GAGTGGATTGTTAGAATGCGC |
| CX3 | *C99TGT1*^1^ | (TGT)_6_ | 214 | FAM | F: GCTGTCATCGCCGAAGAAGT |
|  |  |  |  |  | R: GCAACCACAACCACAAGTCG |
| CX4 | *CxpGT9*^2^ | (GT)_13_ | 108 - 142 | FAM | F: AATCTCCCCGTATAATTGTG |
|  |  |  |  |  | R: TATAAGACCAGTGAAGCCAG |
| CX5 | *CxpGT12*^2^ | (TG)_14_ | 144 - 178 | FAM | F: AACGTGAGCGTGATTGCTC |
|  |  |  |  |  | R: CAGCTGTTGCACCAATGTC |
| CX6 | *CxqATG9*^3^ | (ATG)_7_ | 184 - 262 | FAM | F: CCACTCAAACTAAAACACCACA |
|  |  |  |  |  | R: AATGCCATAACCATCGTCAT |
| CX7 | *C205TG1*^1^ | (TG)_12_ | 150 | HEX | F: CGGTTGACTTTTCGTCGCTGT |
|  |  |  |  |  | R: CATGGGCCACGGTCATATCC |
| CX8 | *C134AC1*^1^ | (AC)_7_ | 195 | HEX | F: TGAAGGTCAGCCACTCAGGC |
|  |  |  |  |  | R: ACAGCTGACTCTCGTCGACA |
| CX9 | *C446AC2*^1^ | (AC)_7_ | 256 | HEX | F: GTGAAGTGGGTGAAGATTAGC |
|  |  |  |  |  | R: CGCGTTTATTCCGGCTTCG |
| CX10 | *CxpGT51*^2^ | (TG)_4_CG(TG)_15_ | 108 - 174 | HEX | F: GAGTATCGCTCGTTGGAGATT |
|  |  |  |  |  | R: ACCCTCTTTTCTTTCTATGTCTGT |
| CX11 | *CxqGT4*^4^ | (GT)_12_ | 152 - 156 | HEX | F: GAACTTGTTCGCCGTCTCAG |
|  |  |  |  |  | R: ATAGAACTTGTTCGCCGTCTC |
| CX12 | *CxpGT46*^2^ | (TG)_15_ | 260 - 286 | HEX | F: CCGACACCGTGTTCAAAGAG |
|  |  |  |  |  | R: TGACGACGACGGTACAAGAG |
| CX13 | *C68GA1*^1^ | (GA)_8_ | 154 | NED | F: ACACGTGGTGCGATGATCC |
|  |  |  |  |  | R: ATCAGCTGATGGTAACCCAGA |
| CX14 | *C139TG1*^1^ | (TG)_10_ | 201 | NED | F: GAGCTGTCATTCTTGGAGGC |
|  |  |  |  |  | R: CGTCCATTTTTCCACGTTCGAC |
| CX15 | *C65AC1*^1^ | (AC)_13_ | 305 | NED | F: GGAGTTGTGCGGTTGAAAGT |
|  |  |  |  |  | R: GCACTGCCTAACGGATCATT |
| CX16 | *C48GTT1*^1^ | (GTT)_6_ | 328 | NED | F: GTGGGTCACTTCGAAGGACACC |
|  |  |  |  |  | R: CTGGGAACCGTTGGCTGATCC |
| CX17 | *CxqTRi4*^4^ | (TGC)_7_ | 120 - 123 | NED | F: CTAGCCCGGTATTTACAAGAAC |
|  |  |  |  |  | R: AACGCCAGTAGTCTCAGCAG |
| CX18 | *CxqCAG101*^3^ | (CAG)_6_ | 174 - 249 | NED | F: CAATCAGGGAACCTCAATC |
|  |  |  |  |  | R: GGGACTGGGTATTAGGAGAC |

^1^Hickner et al. (2010)

^2^Keyghonbadi (2004)

^3^ Edillo et al. (2007)

^4^Smith et al. (2005)
