# Supplementary material for: Effective population size of Culex quinquefasciatus under insecticide-based vector management and following Hurricane Harvey in Harris County, Texas
Source: Front Genet. 2023 Nov 22;14:1297271. doi: 10.3389/fgene.2023.1297271 (PMC10702589; doi:10.3389/fgene.2023.1297271)
Supplement: Supplementary file 3 [file Table3.DOCX]

Supplementary Table 3. N_e_ parameter estimates generated from the most probable scenario for the winter season with a prior distribution of Uniform distribution.

|  |  | | Posterior Effective Population Sizes | | | |
| --- | --- | --- | --- | --- | --- | --- |
| Area | | Parameter | | Median | Mode | 95% HDP^1^ |
| 415 | | N_summer16u_ | | 523,000 | 287,000 | 86,200 – 973,000 |
|  | | N_winter16u_ | | 93,800 | 4,150 | 5,210 – 195,000 |
| 802 | | N_summer16u_ | | 541,000 | 254,000 | 93,400 – 976,000 |
|  | | N_spring17u_ | | 97,300 | 8,520 | 6,140 – 195,000 |

^1^HDP = Highest Posterior Density
