# Supplementary material for: Effective population size of Culex quinquefasciatus under insecticide-based vector management and following Hurricane Harvey in Harris County, Texas
Source: Front Genet. 2023 Nov 22;14:1297271. doi: 10.3389/fgene.2023.1297271 (PMC10702589; doi:10.3389/fgene.2023.1297271)
Supplement: Supplementary file 4 [file Table4.DOCX]

Supplementary Table 4. Time parameter estimates generated from the most probable scenario for the winter season with a prior distribution of Uniform distribution.

|  |  | | Posterior Population Size Changing Time (Measured by Generation) | | | |
| --- | --- | --- | --- | --- | --- | --- |
| Area | | Parameter | | Median | Mode | 95% HDP^1^ |
| 415 | | T_11u_ | | 7.2 | 0.89 | 0 – 15.0 |
| 802 | | T_12u_ | | 9.6 | 0.91 | 0.54 – 20.0 |

^1^HDP = Highest Posterior Density
